# The Effect of Bone Marrow Mesenchymal Stem Cells on the Granulocytic Differentiation of HL-60 Cells

**DOI:** 10.4274/tjh.2016.0498

**Published:** 2018-03-06

**Authors:** Hossein Nikkhah, Elham Safarzadeh, Karim Shamsasenjan, Mehdi Yousefi, Parisa Lotfinejad, Mehdi Talebi, Mozhde Mohammadian, Farhoud Golafshan, Aliakbar Movassaghpour

**Affiliations:** 1 Tabriz University Faculty of Medicine, Hematology and Oncology Research Center, Tabriz, Iran; 2 Tabriz University Faculty of Medicine, Drug Applied Research Center, Tabriz, Iran; 3 Tabriz University Faculty of Medicine, Department of Immunology, Tabriz, Iran; 4 Mazandaran University Faculty of Medicine, Amol Faculty of Paramedical Sciences, Sari, Iran; 5 Hamline University Faculty of Medicine, Department of Biology, Minnesota, USA

**Keywords:** Mesenchymal stem cells, HL-60 cells, Differentiation, All-trans-retinoic acid

## Abstract

**Objective:**

Mesenchymal stem cells (MSCs) are multipotent stromal cells that can differentiate into a variety of cell types. They control the process of hematopoiesis by secreting regulatory cytokines and growth factors and by the expression of important cell adhesion molecules for cell-to-cell interactions. This investigation was intended to examine the effect of bone marrow (BM)-derived MSCs on the differentiation of HL-60 cells according to morphological evaluation, flow cytometry analysis, and gene expression profile.

**Materials and Methods:**

The BM-MSCs were cultured in Dulbecco’s modified Eagle’s medium supplemented with 10% fetal bovine serum (FBS). After the third passage, the BM-MSCs were irradiated at 30 Gy. To compare how the HL-60 cells differentiated in groups treated differently, HL-60 cells were cultured in RPMI-1640 and supplemented with 10% FBS. The HL-60 cells were seeded into six-well culture plates and treated with all-trans-retinoic acid (ATRA), BM-MSCs, or BM-MSCs in combination with ATRA, while one well remained as untreated HL-60 cells. The expression levels of the granulocyte subset-specific genes in the HL-60 cells were assayed by real-time polymerase chain reaction.

**Results:**

Our results revealed that BM-MSCs support the granulocytic differentiation of the human promyelocytic leukemia cell line HL-60.

**Conclusion:**

Based on the results of this study, we concluded that BM-MSCs may be an effective resource in reducing or even preventing ATRA’s side effects and may promote differentiation for short medication periods. Though BM-MSCs are effective resources, more complementary studies are necessary to improve this differentiation mechanism in clinical cases.

## Abstract (tr)

**Amaç:**

Mezankimal kök hücreler (MKH) birçok hücre tipine göre farklılaşabilen multipotent stromal hücrelerdir. Hematopoez sürecini düzenleyici sitokinler ve büyüme faktörleri salınımı ile ve hücreler arası etkileşim için önemli hücresel adezyon moleküllerinin ifadesi yoluyla kontrol ederler. Bu çalışma da kemik iliği (Kİ) kaynaklı MKH HL-60 hücrelerinin farklılaşması üzerine etkisini morfolojik değerlendirme, akım sitometri ve gen ifade analizi yöntemleriyle araştırılması amaçlanmıştır.

**Gereç ve Yöntemler:**

Kİ-MKH %10 fetal sığır serumu (FSS) içeren Dulbecco’nun modifiye Eagle ortamında kültür edildi. Üçüncü pasaj sonrası, Kİ-MKH 30 Gy ile ışınlandı. HL-60 hücrelerinin farklı şartlarda nasıl farklılaştığını karşılaştırmak için HL-60 hücreler %10 FSS eklenmiş RPMI-1640 ortamında kültür edildi. HL-60 hücreleri altı kuyucuklu plaklarda all-trans retinoik asit (ATRA), Kİ-MKH ve ATRA ile birlikte Kİ-MKH ile muamele edilirken, bir kuyucuğa sadece HL-60 hücreleri kondu. HL-60 hücrelerinde granülosit alt gruplarına özgü genlerin ifade düzeyleri gerçek zamanlı polimeraz zincir reaksiyonu ile değerlendirildi.

**Bulgular:**

Sonuçlarımız Kİ-MKH’nin insan promiyelositik lösemi hücre dizisi HL-60’ın granülositik farklılaşmasını desteklediğini gösterdi.

**Sonuç:**

Bu çalışmanın bulgularına göre, Kİ-MKH’nin ATRA yan etkilerini azaltıcı ve hatta önleyici etkili bir kaynak olduğu ve kısa ilaç kullanımı süreçlerinde farklılaşmayı uyarabileceği sonucunu çıkarttık. Kİ-MKH etkili bir kaynak olsa da, klinik olgularda bu farklılaşma mekanizmasını iyileştirmek için destekleyici ek çalışmalara ihtiyaç vardır.

## Introduction

There are different cell types of the osteoblast lineage in bone and the bone marrow, the most primitive of them being the mesenchymal stem cells (MSCs) [1,2]. MSCs can differentiate into several types of cells and produce important growth factors and cytokines [3,4]. MSCs are defined by the International Society of Cellular Therapy based on three properties: the adherence to plastic in standard culture; the expression of CD105, CD73, and CD90 and lack of expression of CD45, CD34, CD14 or CD11b, CD79a or CD19, and HLA class II; and differentiation potential into osteocytes, adipocytes, and chondrocytes [5,6]. These cells are involved in the regulation of hematopoietic precursor cell proliferation and differentiation [7,8].

All-trans-retinoic acid (ATRA) has a potential role in treating acute myeloid leukemia (AML) and some hematological disorders [9]. It has been recognized that ATRA induces the differentiation of myeloid leukemic cells through growth inhibition [10]. Many studies have reported severe adverse effects of ATRA. Therefore, novel therapeutic strategies need to be developed to decrease ATRA’s potential side effects and enhance the efficacy of this drug. One possible approach is the use of ATRA-based combinations that are more efficient than the single components [11,12,13]. The roles of the various cells in the bone marrow niche are unclear in the differentiation of hematopoietic stem cells, and MSCs, as the precursors of the cellular components, are important cells of the bone marrow niche [14]. To understand the precise interaction between MSCs and leukemic cells, in the current study we investigated whether MSCs affect the differentiation of HL-60 cells.

## Materials and Methods

### Cell Culture

Human promyelocytic leukemia cell line HL-60 (a kind gift from Dr. Abroun, Tarbiat Modares University, Tehran, Iran) was cultured in RPMI-1640 medium (Sigma-Aldrich, St. Louis, MO, USA) supplemented with 10% fetal bovine serum (HyClone, Logan, UT, USA), 100 U/mL penicillin, and 100 µg/mL streptomycin (Sigma, St. Louis, MO, USA). The BM-MSCs (Stem Cell Technology, Tehran, Iran) were cultured in low-glucose Dulbecco’s modified Eagle medium (GIBCO BRL, Gaithersburg MD, USA) containing 10% fetal bovine serum.

### Co-culture Experiments

HL-60 cells (10^5^ cells/mL) were seeded onto plates and treated with ATRA at a concentration of 5x10^-7^ M (Sigma-Aldrich) for 48 h. The co-culture experiments were performed in six-well plates including the HL-60 cells treated with BM-MSCs or those treated with BM-MSCs and 5x10^-7^ M ATRA together. Before co-culturing with cancer cells, the BM-MSCs were irradiated at 30 Gy when they reached 60% confluence. The HL-60 cells came into direct contact with the BM-MSCs.

### Morphological Study of Differentiated Granulocyte Cells

To study the morphological changes, the HL-60 cells were treated with ATRA, BM-MSCs, or a combination of ATRA and BM-MSCs. After 48 h of incubation, the cells were stained with Wright-Giemsa stain and studied by light microscope.

### Flow Cytometric Assessment of Granulocytic Markers for Differentiation

The HL-60 cells (1x10^6^) of the different groups, the co-culture of the HL-60 cells with BM-MSCs, the HL-60 cells with BM-MSCs and ATRA in combination, the HL-60 cells with ATRA as a positive control, and the HL-60 cells without additions as a negative control were harvested and incubated with FITC-labeled anti-CD11b (Becton Dickinson, San Jose, CA, USA) for 30 min at 4 °C. The cells were then analyzed for the evaluation of CD11b expression (a myeloid differentiation marker) with a flow cytometer (Becton Dickinson).

### Real-Time Polymerase Chain Reaction

The expression of the granulocyte subset-specific genes in the treated HL-60 cells was investigated by real-time polymerase chain reaction (RT-PCR) after an incubation period of 48 h. Total RNA was extracted using the QIAzol lysis reagent (QIAGEN, Germantown, MD, USA) according to the manufacturer’s instructions. The cDNA was prepared according to the instructions of the Revert Aid Single Strand Kit (Fermentas, Burlington, ON, Canada). The mRNA levels of PU.1, CD11b, lysozyme, C/EBP-ALPHA, C/EBP-BETA, C/EBP E, MPO, CD64, CD16, GCSFR, and cathepsin G were analyzed using qRT-PCR. The *GAPDH* gene was used as an internal control (Table 1).

### Statistical Analysis

Data were reported as mean ± standard deviation and were analyzed using Graph Pad Prism v 5.00 (Graph Pad Software, Inc., La Jolla, CA, USA). Student’s t-test for single comparisons and two-way ANOVA for multigroup comparisons were used for analysis and p<0.01 was regarded as denoting statistical significance.

## Results

### Flow Cytometry Confirmation of the Nature of the BM-MSCs

**Figure 1:**
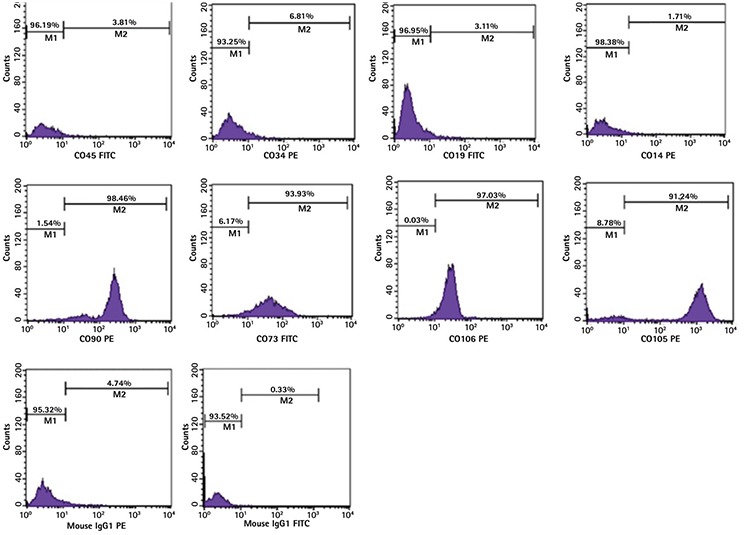
Flow cytometry analysis confirmed the mesenchymal nature of the bone marrow mesenchymal stem cells. The markers assessed by flow cytometry included CD14, CD19, CD34, CD45 CD90, CD105, and CD73. The experiments were done in triplicate.

To verify the mesenchymal nature of the BM-MSCs, the surface antigens were assessed by flow cytometry, including CD14, CD19, CD34, CD45 CD90, CD105, and CD73. The characterization experiments performed in our study demonstrated that the BM-MSCs were negative in the expression of the hematopoietic markers for CD14, CD19, CD34, and CD45, and they had positive expression for CD90, CD105, and CD73 markers (Figure 1).

### Morphological Changes of the Treated Cells

**Figure 2:**
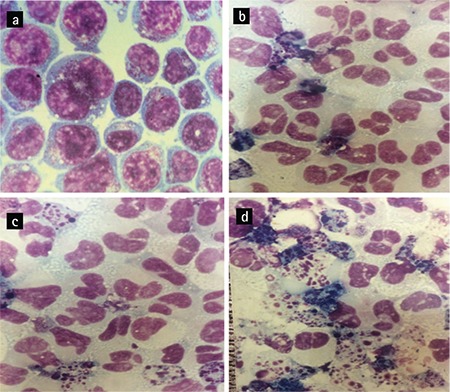
BM-MSCs induced the granulocytic differentiation of HL-60 cells after 48 h of incubation and showed an additive effect with all-trans-retinoic acid (ATRA). The differentiation of the HL-60 cells was assessed by Wright-Giemsa staining: a) untreated HL-60 cells, b) HL-60 cells treated with ATRA, c) HL-60 cells treated with bone marrow mesenchymal stem cells, d) HL-60 cells treated with ATRA and BM-MSCs. Magnitude: 100^x^.

To assess the morphological changes in the treated HL-60 cells, Wright-Giemsa staining was performed (Figures 2A-2D). The comparative study of the morphological changes in the HL-60 cells stained by Wright-Giemsa indicated that, in comparison to the control, the cells treated with ATRA and BM-MSCs individually had induced granulocytic differentiation of the HL-60 cells (Figures 2B and 2C) and showed an additive effect when used with BM-MSCs in combination with ATRA (Figure 2D). While the control cells (Figure 2A) demonstrated typical morphology in the promyelocytic cells (a circular nucleus), the treated HL-60 cells exhibited a kidney-shaped nucleus and segmented nucleus and also had a reduced nuclear/cytoplasmic ratio.

### CD11b Expression Increased in Treated HL-60 Cells

**Figure 3:**
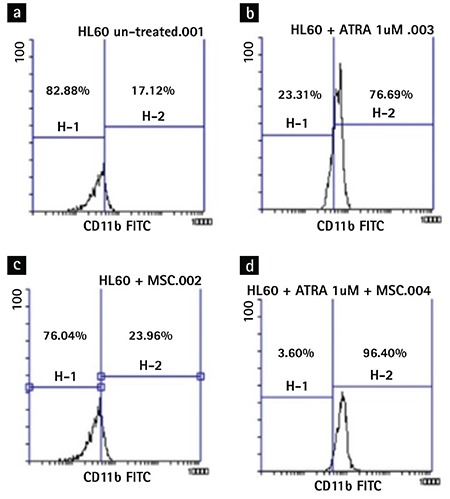
The flow cytometric analysis of CD11b, a granulocytic differentiation marker, after 48 h: a) untreated HL-60 cells, b) HL-60 cells treated with BM-MSCs, c) HL-60 cells co-cultured with all-trans-retinoic acid (ATRA), d) HL-60 cells treated with BM-MSCs and ATRA. BM-MSCs and ATRA synergistically upregulated CD11b expression in cells treated with the combination of the two. The experiments were done in triplicate.

In the treated HL-60 cells, an increase was observed in the percentage of CD11b marker expression, one of the main granulocytic differentiation markers measured by flow cytometry, after 48 h. Flow cytometry results displayed that the expression of the CD11b marker was 17.12%, 76.69%, 23.96%, and 96.4% in the untreated HL-60 cells, in the HL-60 cells treated with ATRA, in the HL-60 cells treated with BM-MSCs, and in the HL-60 cells treated with a combination of BM-MSCs and ATRA, respectively (Figure 3). The expression of CD11b significantly increased in the HL-60 cells treated with the combination of BM-MSCs and ATRA compared to the HL-60 cells treated with ATRA alone or with BM-MSCs alone.

### Effects of BM-MSCs and ATRA on Gene Expression in HLA-60 Cells

**Figure 4:**
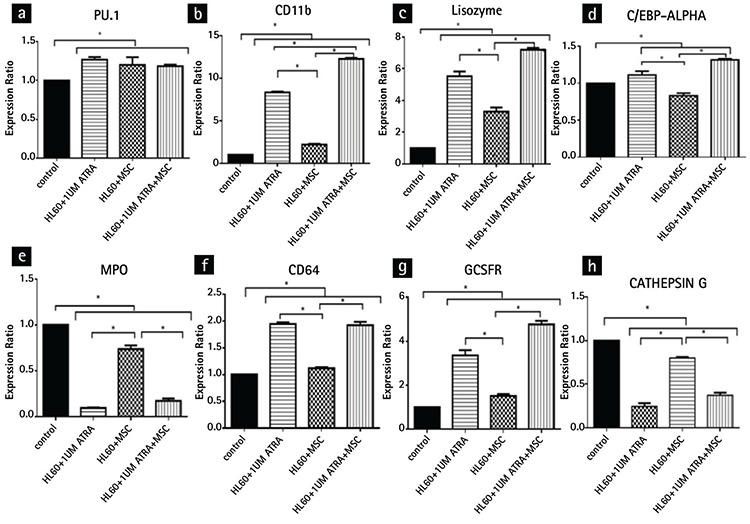
Gene expression during differentiation of the HL-60 cells after 48 h: a) PU.1 gene expression, b) CD11b gene expression, c) lysozyme gene expression, d) C/EBP-alpha gene expression, e) myeloperoxidase gene expression, f) CD64 gene expression, g) GCSFR gene expression, h) cathepsin G gene expression. The experiments were performed in triplicate. *p<0.05. *MPO: Myeloperoxidase, ATRA: all-trans-retinoic acid.*

In the ATRA-treated HL-60 cells, there was a marked increase (p<0.05) in the gene expressions of CD11b, lysozyme, GCSFR, CD64, PU.1, and C/EBP-ALPHA from 1.00 to 8.33 (±0.07), 5.53 (±0.16), 3.36 (±0.12), 1.94 (±0.02), 1.26 (±0.04), and 1.11 (±0.02), respectively. There was no gene expression for C/EBP-BETA, C/EBP E, or CD16 (Figure 4). On the other hand, as revealed in Figure 4, in the HL-60 cells co-cultured with the BM-MSCs, there was significant increase (p<0.05) in CD11b, lysozyme, PU.1, CD64, and GCSFR expression levels from 1.00 to 2.2 (±0.07), 3.3 (±0.16), 1.23 (±0.02), 1.11 (±0.02), and 1.51 (±0.12), respectively, and there was no expression of C/EBP-BETA, C/EBP E, or CD16 levels. In the HL-60 cells co-cultured with the combination of BM-MSCs and ATRA, the gene expression of CD11b, lysozyme, CD64, GCSFR, C/EBP-ALPHA, and PU.1 was markedly increased (p<0.05) from 1.00 to 12.26 (±0.07), 7.19 (±0.16), 1.92 (±0.02), 4.77 (±0.12), 1.31(±0.02), and 1.18 (±0.04), respectively. There was no expression for C/EBP-BETA, C/EBP E, or CD16 (Figure 4). The myeloid differentiation was characterized by downregulation of myeloperoxidase (MPO), a major protein expressed in myeloid cells. We assessed the mRNA level of MPO by RT-PCR after 48 h of treatment. The BM-MSCs, like ATRA, tended to decrease the MPO transcription (Figure 4).

## Discussion

MSCs can support hematopoiesis by producing soluble factor(s) and also by the expression of cell adhesion molecules that are important for cell-to-cell interaction [15]. MSCs have been the subject of particular interest in recent years due their great potential for treating various diseases, especially those related to immune system disorders. However, there are controversial opinions on the role of MSCs in malignancies [16,17,18,19].

In recent years, several groups investigated the possible role of MSCs in influencing the behavior of tumor cells [20,21]. These studies mostly focused on the proliferation and apoptosis of cancer cells, but little is known about the effect of MSCs in the differentiation of leukemic cells [22]. It has been shown that substances such as ursolic acid, 12-O-tetradecanoylphorbol 13-acetate, and 1,25-dihydroxyvitamin D3 [1,25(OH)2D3] inhibit the proliferation of and promote the monocyte/macrophage differentiation of AML HL-60 cells. A secosteroid, 1,25(OH)2D3 has a potential role in the differentiation of the cells of the myeloid lineage in vitro and ex vivo. This ability results in the use of 1,25(OH)2D3 to treat myelodysplastic syndromes or AML. However, 1,25(OH)2D3 leads to the partial differentiation of the hematopoietic blast cells and hypercalcemia, which is a limiting factor in its clinical application [23,24]. Differentiation therapy in APL patients with ATRA alone or in combination with chemotherapy has made great breakthroughs and results in high rates of complete clinical remission. However, it has potentially fatal side effects, such as retinoic acid syndrome and the development of resistance to this drug [13,25]. Repeated treatment with ATRA results in progressive resistance that it is attributed to the decrease of the ATRA serum level, which may be caused by accelerated clearance [26]. The use of ATRA in combination is one possible method to increase the therapeutic efficacy of this drug. Therefore, increasing efforts have been focused on developing alternative differentiation-promoting therapeutic methods with fewer side effects [22]. MSCs possess great advantages in research and clinical applications because of their better expandability, sufficient supply, and painless collection process [27].

Previous studies have shown that ATRA induces morphological differentiation of HL-60 cells. The results from this study indicated that ATRA, BM-MSCs, and ATRA in combination with BM-MSCs promote the differentiation of HL-60 cells compared to untreated cells. It should be added that the HL-60 cells treated with both ATRA and BM-MSCs appeared more mature, presenting band-form nuclei and segmented nuclei, compared to cells treated with either ATRA or the BM-MSCs alone (Figure 2). Matching of the morphological and immunophenotypic data is critical, so immunophenotypic evaluations were performed. The proliferating HL-60 cells, in contrast to monocytes and neutrophils, do not express the CD11b marker, the b-subunit of integrin-aMb2 (also known as CD11b/CD18, MAC-1, or CR3). It was demonstrated that most HL-60 cells, following treatment with D3 (90% at 3-4 days) or ATRA (80% at 4-5 days), become CD11b-positive [28]. As shown in Figure 3, the morphological data were further confirmed by the results of the immunophenotyping of CD11b. After 48 h of treatment, the expression of the CD11b marker in the HL-60 cells co-cultured with BM-MSCs in combination with ATRA was higher than that in the HL-60 cells co-cultured with BM-MSCs or ATRA individually. Therefore, we concluded that BM-MSCs induce the granulocytic differentiation of HL-60 cells.

Our data described the changes in the gene expression pattern during the transformation of the proliferating HL-60 cells into mature cells. One of the important factors that regulate the differentiation of HSCs along the myeloid lineage towards granulocytes rather than monocytes is CCAAT-enhancer binding protein-alpha (C/EBP-ALPHA). Indeed, C/EBP alpha knock-out mice demonstrate an early block in granulocytic differentiation [29]. The results of this study indicate that the BM-MSCs enhance ATRA’s effect on the amplification of C/EBP-ALPHA transcription, but the BM-MSCs alone were upregulated without statistical significance. Our data also showed that the BM-MSCs and ATRA synergistically increased the expression of the CD11b and lysozyme genes.

In this study, we found an increased level of gene expression of PU.1 in the three groups of experiments compared to the untreated cells. Interestingly, we observed no significant synergistic effect in the HL-60 cells treated with ATRA in combination with the BM-MSCs. PU.1 has a critical role in the growth and development of hematopoietic cells. Several studies reported that PU.1-deficient mice lack mature myeloid lineages [30,31]. Uchino et al. [32] reported that the expression of the G-CSF receptor, contrary to their hypotheses, was downregulated after treatment with ATRA. The G-CSF receptor is present in the progenitor cells in the bone marrow, which is involved in the differentiation of the granulocytes through induction of G-CSF [33]. In our study, ATRA upregulated the expression of the G-CSF receptor gene and the use of the BM-MSCs in combination with ATRA synergistically enhanced ATRA’s effect on the expression of this gene, which may demonstrate the critical role of the G-CSF receptor in the promotion of differentiation in promyelocytic leukemia cells. Furthermore, in line with our hypothesis, the treatment with ATRA downregulated the expression of the MPO gene, but the BM-MSCs in combination with ATRA did not have a synergistic effect on the expression of this gene.

## Conclusion

Our results demonstrated that BM-MSCs could promote the granulocytic differentiation of HL-60 cells and could elicit an additive effect when used in combination with ATRA. Consequently, our data highlight the critical role of BM-MSCs in the granulocytic differentiation of HL-60 cells and the use of BM-MSCs and ATRA in combination could be a novel therapeutic strategy for AML patients.

## Figures and Tables

**Table 1:**
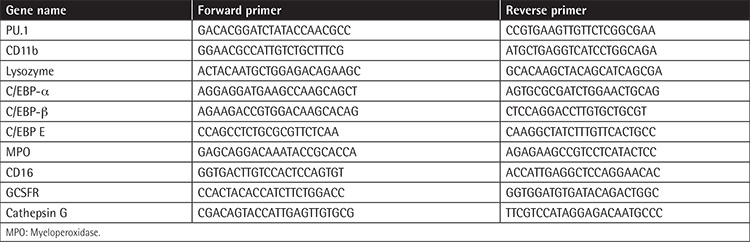
Primers for real-time polymerase chain reaction.
